# Neuropsychiatric disorders in children of mothers with polycystic ovary syndrome: a systematic review and meta-analysis

**DOI:** 10.1186/s12888-026-08047-4

**Published:** 2026-04-04

**Authors:** Qi Cao, Siyu Du, Ziyi Li, Xunxi Liu, Jieyi Zhao, Ziyi Yao, Leyao Qiu, Chunxia Qiao, Huili Zhu, Wei Huang

**Affiliations:** 1 Center for Reproductive Medicine, Department of Gynecology and Obstetrics, West China Second University Hospital, Sichuan University & Key Laboratory of Birth Defects and Related Diseases of Women and Children (Sichuan University), Ministry of Education, Chengdu, 610041 P. R. China; 2 West China School of Medicine, Sichuan University, Chengdu, 610041 P. R. China; 3 Mental Health Center and Psychiatric Laboratory, West China Hospital, Sichuan University, Chengdu, 610041 P. R. China

**Keywords:** Polycystic ovary syndrome, Neuropsychiatric disorders, Offspring, Autism spectrum disorder, Meta-analysis

## Abstract

**Background:**

Polycystic ovary syndrome (PCOS) is a common endocrine disorder among women of reproductive age, yet its association with neuropsychiatric disorders (NPDs) in offspring remains inconsistent and requires updated synthesis.

**Methods:**

We systematically searched PubMed, Embase, Web of Science, and Cochrane Library up to March 2025. A total of 21 observational studies involving 6.8 million mothers and 7.4 million offspring were included. Quality assessment was performed using the Newcastle-Ottawa Scale. Random-effects meta-analyses were conducted to estimate pooled odds ratios (ORs) with 95% confidence intervals (CIs).

**Results:**

Maternal PCOS was significantly associated with increased risks of autism spectrum disorder (OR = 1.44, 95% CI: 1.36–1.53), attention-deficit/hyperactivity disorder (OR = 1.42, 95% CI: 1.35–1.49), chronic tic disorders (OR = 1.45, 95% CI: 1.24–1.68), anxiety (OR = 1.34, 95% CI: 1.27–1.42), other behavioral/emotional disorders (OR = 1.35, 95% CI: 1.30–1.39), and neurological malformations (OR = 1.47, 95% CI: 1.12–1.94). Subgroup analyses by offspring sex and diagnostic criteria showed consistent effects, though sex differences were not significant. Sensitivity analyses confirmed result stability.

**Conclusion:**

This study provides evidence of an association between maternal PCOS and elevated odds of NPDs in children, highlighting the potential importance of prenatal metabolic health and developmental surveillance in this population.

**Clinical trial number:**

Not applicable.

**Supplementary Information:**

The online version contains supplementary material available at 10.1186/s12888-026-08047-4.

## Background

Neuropsychiatric Disorders (NPDs), a diverse group of conditions that affect brain function and behavior, pose a significant global health challenge for children. This category prominently includes autism spectrum disorder (ASD), attention-deficit/ hyperactivity disorder (ADHD), and chronic tic disorders (CTD), which collectively impose substantial developmental burdens. Epidemiological data show that ASD affects 1–2% of children worldwide and its prevalence rates has been steadily increasing, with the number of autism cases per 100,000 people doubling in some regions over the past decade [1–3]; ADHD impacts 2–7% of the global school-aged population [4]; and CTD occurs in 1–2% of pediatric populations [5]. These diseases not only impair children’s cognitive, social, and emotional development [6, 7], but also exert a staggering societal burden. For example, ADHD alone incurs annual costs of $33.2 billion in the U.S. across childhood and adolescence [8].

The etiology of NPDs is multifactorial, involving genetic susceptibility, prenatal environmental insults (particularly adverse in utero exposures), and postnatal environmental factors [9, 10]. Notably, women with PCOS themselves exhibit a higher prevalence of ASD, tics, depressive and anxiety disorders, and ADHD [11, 12]. Since these disorders are known to be partially heritable, this increased maternal prevalence may contribute to the elevated risk observed in their offspring through shared genetic susceptibility [11]. In addition to these genetic components, emerging evidence highlights maternal metabolic disorders during pregnancy as a critical risk factor, with polycystic ovary syndrome (PCOS) emerging as a modifiable and mechanistically compelling risk factor [13–15]. Affecting 5–10% of reproductive-aged women worldwide [16], PCOS is clinically defined by hyperandrogenism, insulin resistance, and chronic inflammation [17, 18]. The Developmental Origins of Health and Disease (DOHaD) theory provides a mechanistic framework: PCOS-induced maternal metabolic dysregulation alters the intrauterine environment, potentially programming long-term neuropsychiatric risk through epigenetic modifications [19, 20]. Experimental evidence indicates maternal hyperandrogenemia may disrupt fetal neural development and neurotransmitter homeostasis, while insulin resistance impairs fetal nutrient metabolism and brain energy supply [15]. These mechanisms converge to suggest a plausible link between maternal PCOS and offspring NPDs. Clarifying the PCOS-NPD association is pivotal for developing prenatal intervention strategies—such as endocrine monitoring and metabolic modulation—to mitigate offspring neurodevelopmental risk.

However, existing research yields conflicting results. While most studies associate maternal PCOS with increased NPD risk in offspring [14, 21], Schieve et al. found no significant association between PCOS and ASD in first-born children [22]. Auyeung et al. discovered fetal testosterone exposure can increase the incidence of ASD [23], while Xu et al. suggested no significant association between high maternal plasma testosterone levels and offspring ASD, possibly because androgen levels in amniotic fluid are also influenced by fetal androgen production [24]. Discrepancies also emerge in gender-specific effects: some studies report stronger associations in female offspring [25], whereas others lack gender-stratified analyses. Methodological variations further complicate interpretations, including divergent PCOS diagnostic criteria (e.g., Rotterdam criteria vs. self-reporting in Bell et al. [26]) and heterogeneous study designs.

To date, the most recent large-scale meta-analysis, published by Dubey et al. in 2021, suggested an association between PCOS and NPDs in offspring [27]. On one hand, a number of new high-quality studies [26–31] have been published up to now, and their results need to be incorporated [28–33]. On the other hand, we also noted discrepancies in the interpretation of primary study data in Dubey’s meta-analysis. For example, in Palomba’s study, none of the offspring included met the diagnostic criteria for ASD [25], and Mamidala et al.‘s study provided data only on hormonally-treated PCOS patients but was incorporated into the overall analysis [34]. Furthermore, Lee’s study and Kosidou’s study overlap in the study population [35, 36].

These gaps underscore the need for updated evidence synthesis to comprehensively synthesize the latest evidence on maternal PCOS and offspring NPDs risk. While neurological malformations are not traditionally classified as neuropsychiatric disorders, they are included in this review as they may reflect early structural alterations in neurodevelopment that could predispose offspring to later functional impairments [37]. Their inclusion broadens the understanding of potential neurodevelopmental consequences associated with maternal PCOS.

Methods.

## Literature search strategy

The protocol for the original review was prospectively registered in PROSPERO (2024 CRD42024615469). Comprehensive searches were conducted in PubMed, Embase, Web of Science and Cochrane library from inception to 31 December 2025 to capture the latest research outcomes. We developed search strategies using Medical Subject Headings (MeSH) and free-text terms across three domains: polycystic ovary syndrome, neuropsychiatric disorders and offspring. To ensure a more comprehensive search, we also included 87 standardised assessment tools as search terms. The complete search syntax for each database is available in Table S1. Additionally, we screened studies included in the previous systematic review by Dubey et al. (2021) to ensure comprehensive evidence coverage [38]. These records underwent identical eligibility assessment as newly identified studies. The reference lists of included studies were reviewed to expand the search scope and avoid missing relevant research. The selection of studies was also limited to articles written in English. Descriptive studies (case series, case reports), clinical trials, non-human studies, abstracts, reviews, and duplicate studies were excluded from this study.

### Inclusion and exclusion criteria

Full articles and abstracts were independently reviewed for the eligibility criteria by two authors (CQ and DY). We established inclusion criteria based on the following key elements: Population: Biological mothers of live-born children; Exposure: polycystic ovary syndrome (PCOS) diagnosed using standardized clinical criteria such as the Rotterdam criteria or NIH criteria, or identified through validated self-report or diagnostic codes in population-based registries; Outcome: neuropsychiatric disorders in offspring, including autism spectrum disorder (ASD), attention-deficit/hyperactivity disorder (ADHD), chronic tic disorders (CTD), anxiety disorders, any other behavioral/emotional disorders, and neurological malformations; Study design: observational studies (cohort studies, case-control studies, and cross-sectional studies). We excluded studies not published in English, those involving only non-biological mothers, and those lacking sufficient data for quantitative synthesis. Specifically, studies were excluded if effect estimates such as odds ratios, risk ratios, or hazard ratios with confidence intervals could not be calculated or extracted.

### Data extraction

Two authors (CQ, DY) independently reviewed each article and extracted relevant data pertaining to this study. To reduce the risk of duplication or bias, an investigator (LX) double checked the compiled data and proofread them for accuracy. Disagreements were resolved by discussion with senior authors (ZL). We extracted data from the studies and categorized the extracted data into four groups: basic information about the study (authors of the study, year, and type of study), basic information about the included mothers (time frame of recruitment, criteria for the PCOS, sample size of the PCOS group versus the control group as well as the total sample size, mean or proportion of the BMI, mean age post proportions in each age group) and basic information about the offspring (sample size with male to female ratio in the PCOS and control groups and total sample size, neuropsychiatric outcomes included, and the age mean or range of assessment) and outcome data (number of cases, number of controls, effect estimates [Odds Ratio (OR), Relative Risk (RR), or Hazard Ratio (HR)] and 95% Confidence Interval (CI)) if applicable.

### Quality assessment

The Newcastle-Ottawa Scale (NOS) was used to evaluate the quality of the included studies by three independent reviewers (DY, LY and LX), which was scored on three dimensions: selection of the study population, between-group comparability, outcome measures for cohort studies and identification of exposure factors for case-control studies [39]. The scale was scored out of 9, with a score of ≥ 7 recognized as a good-quality study, a score of 5–6 recognized as a fair quality study, and a score of ≤ 4 recognized as a poor-quality study according to the Agency for Healthcare Research and Quality (AHRQ) standards [40]. Any discrepancies in scores will be further resolved by a senior author (ZL).

We further evaluated the certainty of evidence for each neuropsychiatric outcome using the Grading of Recommendations Assessment, Development and Evaluation (GRADE) framework [41]. In the GRADE approach, evidence from observational studies starts at a “low” certainty rating by default. The certainty of evidence may be downgraded based on five domains: risk of bias, inconsistency, indirectness, imprecision, and publication bias. Conversely, evidence can be upgraded if there is a large magnitude of effect, evidence of a dose-response gradient, or if all plausible residual confounding would reduce the observed effect [41]. The final certainty of evidence for each outcome was categorized into four levels: high, moderate, low, or very low [41].

### Statistical analysis

The odds ratio(OR) and 95% CI were employed as combined effect metrics to quantify the association between maternal PCOS and offspring NPDs. Since some of the studies included in the meta-analysis reported adjusted associations for various outcomes after adjusting for known risk factors for mothers and children, we used the pooled inverse variance method to combine the effect sizes and estimated the pooled effect size. The DerSimonian and Laird random-effects model was used to obtain a pooled estimate of association. We calculated unadjusted OR based on available frequencies in each study if applicable. Heterogeneity was evaluated using the I² statistic, with thresholds categorized as low (I² <25%), moderate (25–50%), or high (I² >50%).

Subgroup analyses were conducted according to factors including study design (cohort studies vs. case-control studies) and offspring gender (male vs. female). These analyses aimed to explore differences in the association between maternal PCOS and offspring NPDs across subgroups and further identify influencing factors and the p value of subgroup differences in sex were calculated. A leave-one-out sensitivity analysis was performed by sequentially omitting each study to evaluate the reliability and stability of the pooled effect estimates.

All analyses were performed via RevMan 5.4, including forest plots for effect size visualization, funnel plots for publication bias assessment, and subgroup/sensitivity analyses. P values less than 5% were considered statistically significant results.

## Results

### Study selection

**Fig. 1:**
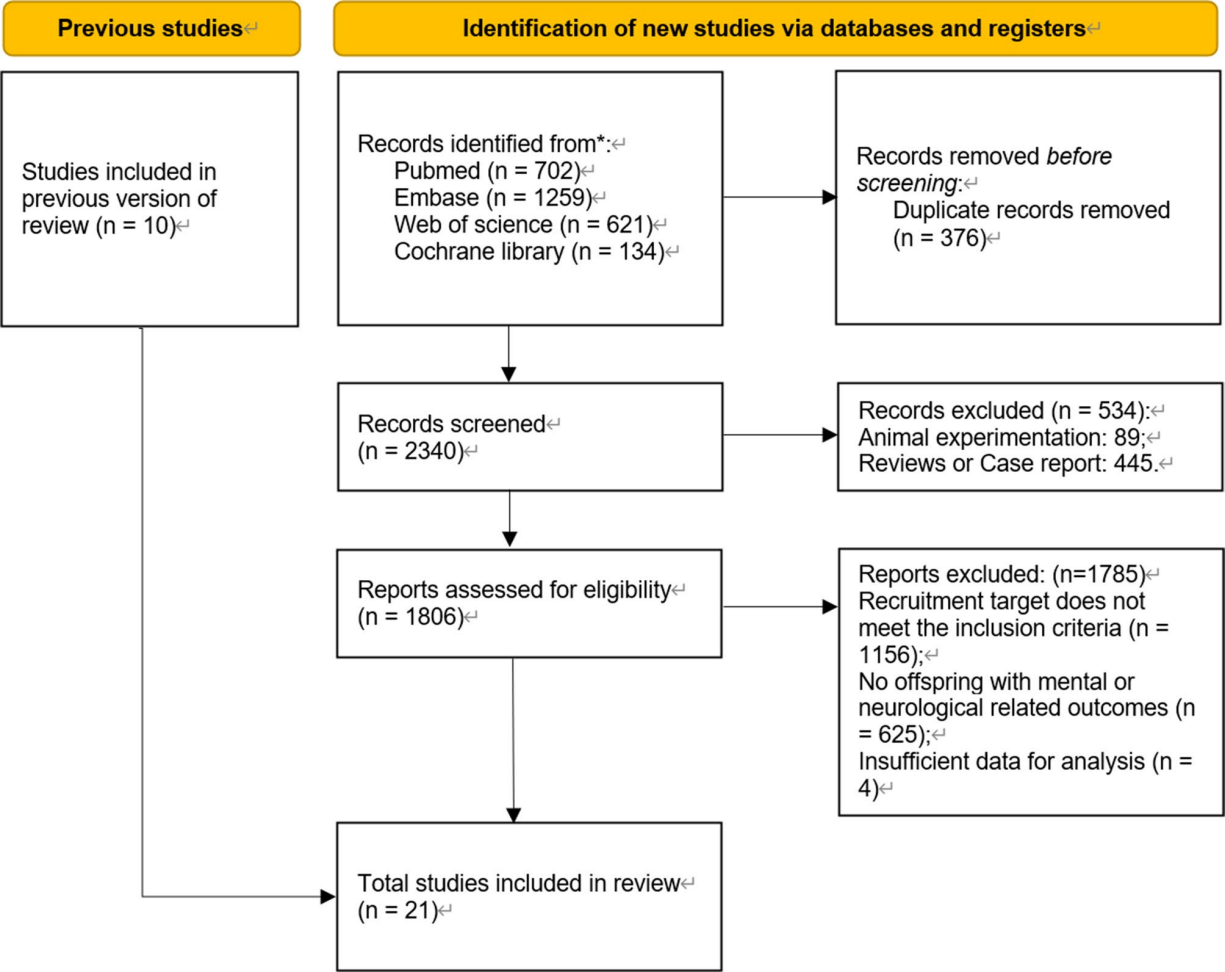
The selection process of studies for meta-analysis. PRISMA flowchart. PRISMA Preferred reporting items for systematic reviews and meta-analyses

Initial searches across PubMed, Embase, Web of Science, and the Cochrane Library yielded 2,716 articles. Following duplicate removal, 2,340 records underwent critical review, of which 1,806 were left for eligible assessment. Ultimately, 21 articles comprising 6,837,312 mothers and 7,425,307 children satisfied all inclusion criteria and were incorporated into quantitative synthesis and quality assessment, with the selection process detailed in the PRISMA flowchart (Fig. 1).

### Study characteristics

Of the 21 included studies, 15 were cohort studies and 7 were case-control studies with a total of 7 outcomes (ASD, ADHD, CTD, anxiety, Ages and Stages Questionnaire (ASQ) domain, other behavioral and emotional disorders and neurological malformations). The screening was considered to have identified a “failure” for a specific ASQ domain if a child’s score fell two standard deviations below the age-specific U.S. national average for that domain [42]. Studies covered multiple countries, including North American countries (one study from Canada, four studies from the United States), Asian countries (four studies from China, one study from Israel), Europe (two studies from the United Kingdom, four studies from Sweden, one study from Finland, two studies from Denmark, and one study from France), and Oceania (two studies from Australia). The time frame for inclusion of patients was from 1973 to 2020. The studies used well-established criteria for PCOS diagnosis, 10 studies used ICD diagnostic criteria, 6 studies used Rotterdam criteria as diagnostic criteria, 4 studies used questionnaires to determine whether patients had polycystic ovary syndrome and 2 studies used read codes as diagnostic basis and one study used both read codes, Rotterdam criteria and national institute of health (NIH) as diagnostic criteria [43]. Read code are a clinical terminology system that was in widespread use in General Practice in the United Kingdom [44]. All the included studies contained 6,839,084 mothers and 7,427,080 offspring. Specific information on mothers and offspring can be found in Table 1. In studies conducting adjusted analysis, maternal age, parental (especially maternal) history of mental illness, prenatal exposures and smaternal body mass index constitute the core set of baseline adjustment variables, covering the vast majority of studies. The detail adjusted variables can be found in Table S3.

**Table 1:** Study characteristics

Author(year)	Country	Study design	Time range of recruiting	Criteria of PCOS inclusion	Total sample size(mothers)	Sample size (PCOS, mothers)	Sample size (Control, mothers)	BMI (kg/m2) mean (SD)/ *n* (%) of mother	Age(year) mean (SD)/ *n* (%) of mother (PCOS/Control)	Total sample size (children)	Sample size (PCOS-child)	Sample size (Control-child)	Male/Female (PCOS-child)	Male/Female (Control-child)	Neuropsychiatric conditions	Age(year) mean(SD)/ range of estimation (PCOS/Control)
Bell, G. A. (2018) [26]	USA	prospective cohort	2008–2010	self-reported	4453	458	3995	< 18.5: 115(2.6); 18.5–25: 2003(45); 25–30: 1138(25.6); > 30: 1192(26.8)	PCOS: 31.2(4.6); Control: 30.3(6.1)	5388: No information for 935 children	458	3995	227/231	1924/2071	ASQ domains	27.5 (12.7)/26.2 (13.2) (month)
Berni, T. R.(2018) [45]	United Kingdom	retrospective cohort	2000–2014	Read code	17,847	8,962	8885	PCOS: 29.86(7.86) Control: 28.99(7.01)	PCOS: 26.90(7.20); Control: 27.01(7.36)	17,847	8962	8885	NA	NA	ADHD, ASD	NA
Cao, Q. (2024) [31]	China	prospective cohort	2019–2020	Rotterdam criteria	91	33	58	PCOS: 21.8(3.1); Control: 20.2(2.0)	NA	91	33	58	NA	NA	ASQ domains	27(month)
Cesta, C. E. (2020) [46]	Sweden	retrospective cohort	1973–2013	ICD	154,376	12,955	141,421	NA	NA	239,099	20,988	218,111	10,779/10,209	103,368/106,643	ADHD, ASD, CTD	9.2(7.6)
Chen, X. (2020) [21]	Finland	retrospective cohort	1996–2014	ICD	590,939	NA	NA	NA	NA	1,097,753	24,682	1,073,071	12,733/11,949	548 533/524,538	ASD, ADHD, CTD, Anxiety	4–22
Cherskov, A. (2018) [43]	United Kingdom	case-control	1990–2014	Read code, NIH, Rotterdam criteria	49,685	8558	41,127	NA	PCOS: 27.7 (5.2); Control: 27.9(5.5)	49,685	8558	41,127	4417/4171	21,098/20,029	ASD	NA
Dalgaard, C. M. (2021) [47]	Denmark	retrospective cohort	2010– 2012	Rotterdam criteria	1772	165	1607	23.7 (21.3; 27.5)/23.3 (21.2; 26.3)	PCOS: 30.8(4.1) Control: 30.4(4.4)	1776	177	1599	88/77	849/762	ADHD	NA
Doherty, D. A. (2015) [48]	Australia	retrospective cohort	1997–2011	ICD	38,361	3505	34,856	NA	< 20: 2563 (6.7); 20–29: 21,266 (55.4); 30–39: 14,209 (37.0); >=40: 323(0.8)	38,966	3626	35,340	NA	NA	Neurological malformations	NA
Fauque, P. (2021) [49]	France	retrospective cohort	2013–2017	ICD	3,508,472	6977	3,501,495	NA	< 20: 79,626 (2.3); 20–29: 1,574,775 (45.0); 30–39: 1,709,422 (48.8); >=40: 137,672 (3.9);	3,508,472	6977	3,501,495	NA	NA	Neurological malformations	2.5
Hisle-Gorman, E. (2018) [50]	USA	case-control	2000–2013	ICD	35,040	556	34,484	NA	NA	35,040	556	34,484	Combined group:27,997/7043		ASD	NA
Jiang, L. (2021) [51]	China	case-control	2015–2016	Rotterdam criteria	61	30	31	NA	PCOS: 32.41 (4.55); Control: 33.66 (3.82)	73	34	39	NA	NA	ASD	3.82(1.25)/3.66(0.59)
Kosidou, K. (2016) [36]	Sweden	case-control	1984–2011	ICD	232,544	1006	231,538	NA	NA	232,544	1006	231,538	737/269	161,902/72,636	ASD	NA
Kosidou, K. (2017) [52]	Sweden	case-control	1984–2011	ICD	558,910	2341	556,569	NA	NA	558,910	2341	556,569	1684/657	381,635/174,934	ADHD	NA
Palm, C. V. B. (2023) [32]	Denmark	retrospective cohort	2010–2012	Rotterdam criteria	1773	165	1608	PCOS: 23.7 (6.4); Control: 23.3 (5.1)	PCOS: 30.8 (4.1); Control: 30.4 (4.4)	1773	165	1608	88/77	846/762	ASD	27 months
Risal, S. (2021) [33]	Sweden	retrospective cohort	1990–2013	ICD	102,466	8864	93,602	NA	< 20: 3193 (3.1); 20–29: 63,570 (62.0); 30–34: 28,472 (27.8); >=35: 7231 (7.1);	102,466	8864	93,602	4574/4290	47,806/45,796	Anxiety	6–18.9
Robinson, S. L. (2020) [53]	USA	Prospective cohort	2008–2010	self-reported	1624	194	1430	26.9(7.0)	31.3(5.9)	1915	NA	NA	Combined group:1017/898		ADHD, Anxiety	7–8
Rotem, R. S. (2021) [54]	Israel	retrospective cohort	1999–2013	ICD	437,222	17,447	419,775	NA	PCOS: 31.2; Control: 31.2	437,222	17,447	419,775	9112/8335	216,031/203,744	ASD	NA
Wang, Y. (2021) [55]	China	case-control	2018–2019	Rotterdam criteria	145	29	116	PCOS: 22.32(3.84); Control: 21.23(2.78)	PCOS: 29.21; Control: 28.41	145	29	116	NA	NA	ASQ domain	21.83/21.45(month)
Wei, S. Q. (2022) [29]	Canada	retrospective cohort	2006–2019	ICD	1,038,375	7160	1,031,215	NA	< 30: 500,030(48.2); 30–34: 350,653(33.8); > 35: 187,692(18.1)	1,038,375	7160	1,031,215	NA	NA	Neurological malformations	NA
Yuying Zhang (2022) [30]	China	Cohort	2021	self-reported	63,390	1,667	61,723	NA	29.4/29.1	63,390	1,667	61,723	33,065/28,658	891/776	ADHD	4.71/4.86
Schieve, L. A.(2017) [56]	USA	case-control	2007–2012	self-reported	1538	79	1459	NA	NA	1538	79	1459	NA	NA	ASD	2–5
Total					6,837,312	80,986	6,165,387			7,425,307	113,644	7,314,210				

Read code was used as a diagnostic classification according to UK primary care practice standard

ASD autism spectrum disorder, ADHD attention deficit hyperactivity disorder, CTD chronic tic disorder, PCOS Polycystic ovary syndrome, NIH national institute of health, ICD international classification of diseases, SD standard deviation, NA not available

## Quality assessment and publication bias

Using the AHRQ standards, most studies were classified as good quality (*n* = 19) or fair quality (*n* = 2) studies (Table S2). The funnel plots for all outcomes were symmetrical in both the unadjusted (Figure S1) and adjusted (Figure S4) analysis, indicating a low risk of publication bias. The GRADE certainty of evidence was rated as low for ASD and ADHD, while for the rest of outcomes it was rated as very low (Table S4).

### Main outcomes

Association between maternal PCOS and ASD in children.

A total of 10 studies were included in the analysis. In adjusted OR analysis, the odds of having ASD in the offspring of patients with PCOS was increased by 44% (OR: 1.44; 95% CI: 1.36, 1.53; *p* < 0.001; I^2^ =0%). The GRADE certainty of evidence for ASD was rated as low. The effect of PCOS on the odds of having ASD in the offspring was statistically significant in all subgroup analyses, with a 43% increased odds of ASD in the offspring of patients with PCOS in the cohort study (OR: 1.43 ; 95% CI: 1.34, 1.53; *p* < 0.001; I^2^ = 0%), and in the case-control studies, the odds of having ASD in the offspring of patients with PCOS was increased by 49% (OR: 1.49 ; 95% CI: 1.29, 1.71; *p* < 0.001; I^2^ = 0%) (Table 2 and Figure S3). The unadjusted OR values can be found in Table 2 and Figure S2. In a subgroup analysis of gender, it was shown that male offspring of pregnant PCOS patients had a 43% increased odds of having ASD (OR: 1.43; 95% CI: 1.31, 1.56; *p* < 0.001; I^2^ = 0%) and female offspring had a 63% increased odds of having ASD (OR: 1.63; 95% CI: 1.37, 1.93; *p* < 0.001; I^2^ = 12%). However, the differences between gender subgroups were not statistically significant (*p* = 0.18)**(**Table 3**)**.

**Table 2:** Association between maternal PCOS and neuropsychiatric disorders in their children

Neuropsychiatric disorders		Unadjusted association				Adjusted association^a^			GRADE
	*N*	OR (95% CI)	*p* value	I^2^(%)		OR (95% CI)	*p* value	I^2^(%)	
**Cohort studies**									
ASD	5	1.42 [1.32, 1.52]	< 0.001*	0		1.43 [1.34, 1.53]	< 0.001*	0	/
ADHD	6	1.30 [1.17, 1.44]	< 0.001*	61		1.42 [1.35, 1.50]	< 0.001*	0	/
CTD	2	1.39 [1.20, 1.61]	< 0.001*	0		1.45 [1.24, 1.68]	< 0.001*	0	/
Anxiety	3	1.34 [1.27, 1.41]	< 0.001*	0		1.34 [1.27, 1.42]	< 0.001*	0	/
ASQ domain (any fail)	3	1.60 [1.16, 2.20]	0.004*	0		1.36 [0.97, 1.90]	0.07	NA	/
Other behavior/emotional problems	1					1.35 [1.30, 1.39]	< 0.001*	20	/
Neurological malformations	3	1.59 [1.31, 1.94]	< 0.001*	69		1.47 [1.12, 1.94]	0.006	86	/
**Case-control studies**									
ASD	5	1.66 [1.44, 1.93]	< 0.001*	38		1.49 [1.29, 1.71]	< 0.001*	0	/
ADHD	1	1.78 [1.60, 1.99]	< 0.001*	NA		1.40 [1.25, 1.56]	< 0.001*	NA	/
**Total**									
ASD	10	1.51 [1.39, 1.65]	< 0.001*	42		1.44 [1.36, 1.53]	< 0.001*	0	Low
ADHD	7	1.38 [1.20, 1.58]	< 0.001*	84		1.42 [1.35, 1.49]	< 0.001*	0	Low
CTD	2	1.39 [1.20, 1.61]	< 0.001*	0		1.45 [1.24, 1.68]	< 0.001*	0	Very low
Anxiety	3	1.34 [1.27, 1.41]	< 0.001*	0		1.34 [1.27, 1.42]	< 0.001*	0	Very low
ASQ domain (any fail)	3	1.60 [1.16, 2.20]	0.004*	0		1.36 [0.97, 1.90]	0.07	NA	Very low
Other behavior/emotional problems	1					1.35 [1.30, 1.39]	< 0.001*	20	Very low
Neurological malformations	3	1.59 [1.31, 1.94]	< 0.001*	69		1.47 [1.12, 1.94]	0.006	86	Very low

N number of studies, ASD autism spectrum disorder, ADHD attention deficit hyperactivity disorder, PCOS Polycystic ovary syndrome, CTD chronic tic disorder, ASQ Ages & Stages Questionnaires, OR odds ratio, CI confidence interval, * p-value < 0.05 indicates statistical significance

^a^Adjusted association if available

**Table 3:** Association of maternal PCOS and NPD in children by their sex

Neuropsychiatric disorders		Unadjusted association					Adjusted association			
	*N*	OR (95% CI)	*p* value	I^2^(%)	*P* value of subgroup differences in sex	*N*	OR (95% CI)	*p* value	I^2^(%)	*P* value of subgroup differences in sex
ASD					0.32					0.18
Male	5	1.45 [1.23, 1.70]	0.01*	69		4	1.43 [1.31, 1.56]	< 0.001*	0	
Female	5	1.61 [1.41, 1.85]	< 0.001*	0		4	1.63 [1.37, 1.93]	< 0.001*	12	
ADHD					0.85					0.19
Male	5	1.39 [1.14, 1.70]	< 0.001*	90		4	1.39 [1.31, 1.47]	< 0.001*	0	
Female	5	1.43 [1.17, 1.74]	< 0.001*	77		4	1.49 [1.36, 1.64]	< 0.001*	0	
CTD					0.68					0.73
Male	2	1.35 [1.14, 1.61]	< 0.001*	0		2	1.42 [1.19, 1.70]	< 0.001*	0	
Female	2	1.45 [1.07, 1.97]	< 0.001*	0		2	1.51 [1.11, 2.05]	0.009*	0	

N number of studies, ASD autism spectrum disorder, ADHD attention deficit hyperactivity disorder, PCOS Polycystic ovary syndrome, CTD chronic tic disorder, ICD International Classification of Disease codes, OR odds ratio, CI confidence interval, * p-value < 0.05 indicates statistical significance

#### Association between maternal PCOS and ADHD in children

A total of 7 studies were included in the analysis. In adjusted OR analysis, the odds of having ADHD in the offspring of patients with PCOS was increased by 42% (OR: 1.42; 95% CI: 1.35, 1.49; *p* < 0.001; I^2^ = 0%). The GRADE certainty of evidence for ADHD was rated as low. The results of the subgroup analyses were all statistically significant, and when subgroup analyses were performed for the type of study, the odds of having ADHD in the offspring of patients with PCOS was increased by 42% (OR: 1.42; 95% CI: 1.35, 1.50; *p* < 0.001; I^2^ = 0%) in cohort studies, and in case-control studies the odds of ADHD in the offspring of patients with PCOS was increased by 40% (OR: 1.40; 95% CI: 1.25, 1.56; *p* < 0.001) (Table 2 and Figure S3). The unadjusted OR values can be found in Table 2. In subgroup analyses by sex, male offspring of patients with PCOS had a 39% increased odds of having ADHD (OR: 1.39; 95% CI: 1.31, 1.47; *p* < 0.001; I^2^ = 0%), and female offspring had a 49% increased odds of having ADHD (OR: 1.49; 95% CI: 1.36, 1.64; *p* < 0.001; I^2^ = 0%). However, the differences between gender subgroups were not statistically significant (*p* = 0.19).(Table 3).

#### Association between maternal PCOS and CTD in children

A total of 2 cohort studies were included in the analysis. In adjusted OR analysis, offspring of patients with PCOS had a 45% increased odds of having CTD (OR: 1.45; 95% CI: 1.24, 1.68; *p* < 0.001; I^2^ = 0%) (Table 2 and Figure S3). The unadjusted OR values can be found in Table 2. The GRADE certainty of evidence for CTD was rated as very low. In the subgroup analysis by sex, male offspring of PCOS patients had a 42% increased odds of having CTD (OR: 1.42; 95% CI: 1.19, 1.70; *p* < 0.001; I^2^ = 0%) and female offspring had a 51% increased odds of having CTD (OR: 1.51; 95% CI: 1.11, 2.05; *p* = 0.009; I^2^ = 0%) (Table 3). However, the differences between gender subgroups were not statistically significant (*p* = 0.73).

#### Association between maternal PCOS and anxiety in children

A total of 2 cohort studies were included in the analysis. In adjusted OR analysis, offspring of patients with PCOS during pregnancy had a 34% increased odds of having anxiety (OR: 1.34; 95% CI: 1.27, 1.42; *p* < 0.001; I^2^ = 0%). The unadjusted OR values can be found in Table 2. The GRADE certainty of evidence for anxiety was rated as very low.

#### Association between maternal PCOS and ASQ failure in children

A total of 3 cohort studies were included in the unadjusted analysis but only Bell’s study was able to be included for adjusted analysis. There was statistically significant odds of any fail in ASQ domains compared to controls in unadjusted analysis(OR: 1.60; 95%CI: 1.17, 2.20; *p* = 0.04; I^2^ = 0%), but the association become non-significant in adjusted analysis (OR: 1.36; 95%CI: 0.97, 1.90; *p* = 0.07). However, PCOS was associated with higher odds of ASQ failure in the fine motor domain in adjusted analyses (OR = 1.69, 95% CI: 1.16, 2.44) (Figure S5).The unadjusted OR values can be found in Table 2. The GRADE certainty of evidence for ASQ failure was rated as very low.

#### Association between maternal PCOS and other behavioral and emotional disorders in children

In adjusted OR analysis, we combined the behavioral and emotional disorders including mood disorders, eating disorders, sleeping disorders, personality disorders, intellectual disabilities and specific developmental disorders in Chen’s study. The result showed that offspring had a 35% increased odds of having other behavioral and emotional disorders (OR: 1.35; 95% CI: 1.30, 1.39; *p* < 0.001; I2 = 20%). The unadjusted OR values can be found in Table 2. The GRADE certainty of evidence was rated as very low.

Association between maternal PCOS and neurological malformations in children A total of 3 cohort studies were included in the analysis. There was statistically significant odds of hospitalization due to nervous system diseases compared to controls in unadjusted analysis (OR: 1.59; 95%CI: 1.31, 1.94; *p* < 0.001; I^2^ = 69%). However, in adjusted OR analysis, the odds of hospitalization due to nervous system diseases in the offspring of patients with PCOS was not significant (OR: 1.42; 95% CI: 0.96, 2.10; *p* = 0.08; I^2^ = 92)(Table 2). The GRADE certainty of evidence for neurological malformations was rated as very low.

### Sensitivity analyses for NPDs in children

The leave-one-out sensitivity analysis confirmed the robustness of our primary findings. For all outcomes, the direction and magnitude of the pooled effect sizes remained consistent upon the exclusion of any single study, and all associations except neurological malformations retained statistical significance (*p* < 0.05). The OR range for ASD being 1.42–1.46, for ADHD 1.41–1.43, for anxiety 1.33–1.52, for CTD 1.42–1.60 and for neurological malformations 1.34–1.69 (Table 4).

**Table 4:** Sensitive analysis of adjusted analysis

Exclude study	OR (95% CI)	*p* value	I^2^(%)
**ASD**			
Berni, T. R.(2018)	1.44 [1.35, 1.52]	< 0.001*	0
Cesta, C. E. (2020)	1.43 [1.33, 1.53]	< 0.001*	0
Chen, X. (2020)	1.46 [1.36, 1.56]	< 0.001*	0
Palm, C. V. B. (2023)	1.44 [1.35, 1.53]	< 0.001*	0
Rotem, R. S. (2021)	1.45 [1.36, 1.55]	< 0.001*	0
Cherskov, A. (2018)	1.44 [1.36, 1.54]	< 0.001*	0
Kosidou, K. (2016)	1.42 [1.34, 1.52]	< 0.001*	0
**ADHD**			
Berni, T. R.(2018)	1.41 [1.35, 1.48]	< 0.001*	0
Cesta, C. E. (2020)	1.41 [1.33, 1.48]	< 0.001*	0
Chen, X. (2020)	1.41 [1.32, 1.51]	< 0.001*	0
Robinson, S. L. (2020)	1.42 [1.35, 1.49]	< 0.001*	0
Yuying Zhang (2022)	1.43 [1.36, 1.50]	< 0.001*	0
Kosidou, K. (2017)	1.42 [1.35, 1.50]	< 0.001*	0
**Anxiety**			
Chen, X. (2020)	1.52 [1.22, 1.89]	< 0.001*	0
Risal, S. (2021)	1.33 [1.26, 1.41]	< 0.001*	0
Robinson, S. L. (2020)	1.34 [1.27, 1.41]	< 0.001*	0
**CTD**			
Cesta, C. E. (2020)	1.42 [1.21, 1.67]	< 0.001*	NA
Chen, X. (2020)	1.60 [1.08, 2.37]	0.02	NA
**Neurological malformations**			
Doherty, D. A. (2015)	1.69 [1.47, 1.95]	< 0.001*	0
Fauque, P. (2021)	1.42 [0.96, 2.10]	0.08	92
Wei, S. Q. (2022)	1.34 [0.99, 1.82]	0.06	80

ASD autism spectrum disorder, ADHD attention deficit hyperactivity disorder, CTD chronic tic disorder, OR odds ratio, CI confidence interval, * p-value < 0.05 indicates statistical significance

## Discussion

This systematic review and meta-analysis, encompassing 21 studies with data from over 6.8 million mothers and 7.4 million offspring, provides robust evidence that maternal PCOS is significantly associated with an increased risk of a broad spectrum of neuropsychiatric disorders (NPDs) in children. Compared to offspring of mothers without PCOS, those exposed to maternal PCOS exhibited substantially elevated risks for autism spectrum disorder (ASD), attention-deficit/hyperactivity disorder (ADHD), chronic tic disorders (CTD), anxiety disorders, other behavioral/emotional disorders, and neurological malformations. These associations remained consistent across diverse study designs and different gender for PCOS. Although the average effect size for the risk of ASD, ADHD, and CTD in female offspring was higher than that in male offspring, the differences between the gender subgroups were not statistically significant. The stability of these findings was confirmed through sensitivity analyses. The robustness of our findings is supported by the consistent adjustment for key confounders across the included studies (Table S3). Most primary research accounted for maternal age, BMI, and socioeconomic status. Crucially, by adjusting for parental history of neuropsychiatric disorders, these studies minimize the likelihood that the observed risks are merely due to shared genetic susceptibility. This rigorous control reinforces the reliability of the independent association between maternal PCOS and offspring neuropsychiatric outcomes.

In this study, we presented both unadjusted and adjusted effect estimates to evaluate the impact of confounding on the observed associations. Overall, the adjusted effect sizes were slightly attenuated compared to the unadjusted ones, but most outcomes retained statistical significance. This attenuation suggests that a portion of the crude association is explained by potential confounders commonly adjusted for across studies, such as maternal age, BMI, smoking, and history of psychiatric disorders (Table S3). For instance, the association between maternal PCOS and any ASQ domain failure was significant in the unadjusted analysis but lost significance after adjustment, indicating that this specific finding might be particularly susceptible to confounding or may be driven by a single large study. Furthermore, the high heterogeneity observed in the unadjusted analysis for ADHD (I² = 84%) was completely resolved in the adjusted analysis (I² = 0%). This demonstrates that controlling for key confounders, like parental psychiatric history and socioeconomic status, accounts for much of the variability between primary studies, leading to a more consistent and reliable pooled estimate. These observations underscore the critical importance of rigorous confounding control in observational research and suggest that unadjusted models may overestimate the true strength of the association. However, the consistent direction of effects across both unadjusted and adjusted models reinforces the robustness of the finding that maternal PCOS may be an independent, albeit modest, risk factor for offspring neuropsychiatric disorders.

Our findings are generally consistent with Dubey et al.‘s meta-analysis published in 2021 regarding ASD/ADHD risk associations [38], but there are some differences in the studies included. We did not include data from Bell, G. A. (2018), Palomba, S. (2012), and Lee (2017) in the ASD analysis, although these three studies had been incorporated into ASD analyses in Dubey’s article [26, 57, 58]. Because we found that Bell’s study focused on assessment of developmental screening (ASQ) domains rather than clinical ASD diagnoses. Lee’s study included offspring of mothers with hirsutism recruited by Statistics Sweden and the National Board of Health and Welfare in Sweden. The study population overlaps with that of another study conducted by Kosidou et al. [36]. Since not all PCOS patients present with hirsutism, and Kosidou’s study included a more comprehensive range of PCOS patients, we decided to include Kosidou’s study while excluding Lee’s study, because duplicate analysis may introduce bias in the results. In Palomba’s study, none of the offspring’s Autism-Spectrum Quotient (AQ-C) scores exceeded 76 points, meaning none met the diagnostic criteria for ASD, so we did not include this article [59]. After excluding these studies and including new studies published after March 30, 2021, the adjusted effect size of the risk of ASD was slightly higher than that of the previous meta-analysis (OR: 1.44 vs. 1.40). Concurrently, in studies on the impact of PCOS on offspring with ADHD, we included more recent evidence published post-2021, including Zhang et al.‘s large-scale study and Dalgaard’s study [30, 47]. These adjustments yielded more rigorous effect estimates, reinforcing PCOS as an independent NPD risk factor beyond earlier approximations.

Importantly, our analysis extends the clinical spectrum of PCOS-related neurodevelopmental sequelae. Beyond confirming ASD/ADHD links, we also establish associations with CTD, anxiety, other behavioral/emotional disorders, and neurological malformations—a finding absent from prior meta-analyses. Supporting this, all three relevant studies confirmed that maternal PCOS significantly increases the risk of neurological abnormalities in offspring or associated hospitalizations [28, 29, 48].

In addition, this study also conducted an analysis specifically on ASQ scores for the first time. We found that in the unadjusted analysis, offspring of mothers with PCOS had a higher risk of failing any ASQ domain (OR: 1.60) but attenuated in the adjusted analysis. However, PCOS was associated with higher odds of ASQ failure in the fine motor domain in adjusted analyses (OR = 1.69). Given the dominance of one large study (Bell et al.) [26] in this pooled analysis, confirmation in larger, independent cohorts is crucial.

In the gender subgroup analysis of this study, although we observed that the average risk effects for ASD, ADHD, and CTD were slightly higher in female offspring than in male offspring, we did not find any statistically significant differences in the risk effects for these neuropsychiatric disorders between offspring of different genders. And even though previous studies have reported sex differences, results have been inconsistent [60, 61]. Furthermore, the subgroup results in the studies we included were also inconsistent. Some studies found that the effect size in female offspring was greater than that in male offspring [21, 36, 46, 52], while others found that the effect size in male offspring was greater than that in female offspring [43, 62, 63]. Therefore, more and larger studies are needed to investigate the impact of PCOS on the neuro-developmental health of offspring of different sexes.

As a metabolic disorder, weight management plays a pivotal role in the comprehensive management of PCOS. While maternal obesity, common in PCOS, is independently associated with increased offspring NPD risk through mechanisms like chronic low-grade inflammation, hyperglycemia, and insulin resistance [64–67], evidence suggests that PCOS itself contributes independently. Chen et al. [68] demonstrated that even among lean mothers, PCOS is associated with a higher risk of specific offspring NPDs (anxiety, specific developmental disorders, ASD, intellectual disabilities, behavioral/emotional disorders), indicating an effect beyond maternal BMI. Notably, when PCOS co-occurs with obesity, the risks appear further amplified, highlighting the potential for synergistic detrimental effects on the fetal neurodevelopmental environment [68]. However, due to limitations in the current research data, it has not been possible to conduct a stratified analysis of BMI.

Prenatal androgen excess represents the most compelling mechanistic pathway linking maternal PCOS to offspring NPD risk. Beyond epidemiological associations, human and animal evidence converges to demonstrate that fetal hyperandrogenism—driven by maternal endocrine dysfunction and compounded by placental dysregulation—directly disrupts neurodevelopment. Critically, placental tissue in PCOS exhibits reduced aromatase activity (limiting androgen-to-estrogen conversion) while increasing steroidogenesis, thereby amplifying fetal testosterone exposure [69, 70]. This androgen surge impairs synaptic maturation in key brain regions, evidenced by: (1) Persistent alterations in frontal cortex spine dynamics (aberrant formation/elimination in mice, mirroring synaptic pathology in ASD) [71]; (2) Disrupted dopaminergic circuitry and hippocampal dysmaturation [72–75]. These structural changes manifest functionally as autistic-like behaviors in prenatally androgenized animals [76, 77] and align with human neurodevelopmental trajectories. Furthermore, placental FOS-mediated dysregulation of neurotrophic factors provides a molecular bridge between maternal PCOS and female-specific neural vulnerability [78].

Beyond androgen excess and metabolic disturbances, maternal thyroid dysfunction represents a plausible link between PCOS and offspring neuropsychiatric risk as highlighted by recent review [79]. PCOS is frequently associated with an increased prevalence of thyroid disorders, such as autoimmune thyroiditis and subclinical hypothyroidism [80]. Thyroid hormones are essential for critical fetal brain processes, including neuronal migration, differentiation, and myelination. Suboptimal maternal thyroid function has been consistently associated with cognitive delays and an elevated risk of ADHD and ASD in offspring [81, 82]. However, none of the studies currently included in our meta-analysis provided specific data or analyses regarding maternal thyroid hormone levels. This highlights a significant gap in the existing literature, and further well-designed experimental studies are urgently needed to investigate the independent and synergistic effects of thyroid hormones on offspring neurodevelopment in the context of PCOS.

The association between maternal polycystic ovary syndrome (PCOS) and elevated risks of neuropsychiatric disorders (NPDs) in offspring, as demonstrated by our meta-analysis, necessitates a nuanced understanding of the roles played by subfecundity and infertility treatments [79]. PCOS is well-recognized as the most frequent cause of anovulatory infertility, leading a substantial proportion of these patients to utilize assisted reproductive technologies (ART), such as in vitro fertilization (IVF) and intrauterine insemination (IUI) [83]. This clinical reality raises the critical question of whether the observed neurodevelopmental risks—including ASD and ADHD found in our study —are inherent to the maternal PCOS condition or are mediated by the procedures of infertility treatment.

Evidence from a large-scale longitudinal study by Fauque et al. (2021) suggests that underlying maternal infertility factors, including PCOS, contribute an independent risk to offspring development [49]. Their findings revealed that PCOS was associated with a significantly increased risk of congenital anomalies, with neurological malformations being notably more common in children of mothers with infertility regardless of the mode of conception [49]. Crucially, while IVF itself was associated with a moderate increase in risks, these were partly attributed to the cumulative effect of the underlying maternal subfecundity. This supports the hypothesis that the maternal biological environment in PCOS—characterized by metabolic and endocrine dysregulation—is a primary driver of adverse neurohealth outcomes.

**Fig. 2:**
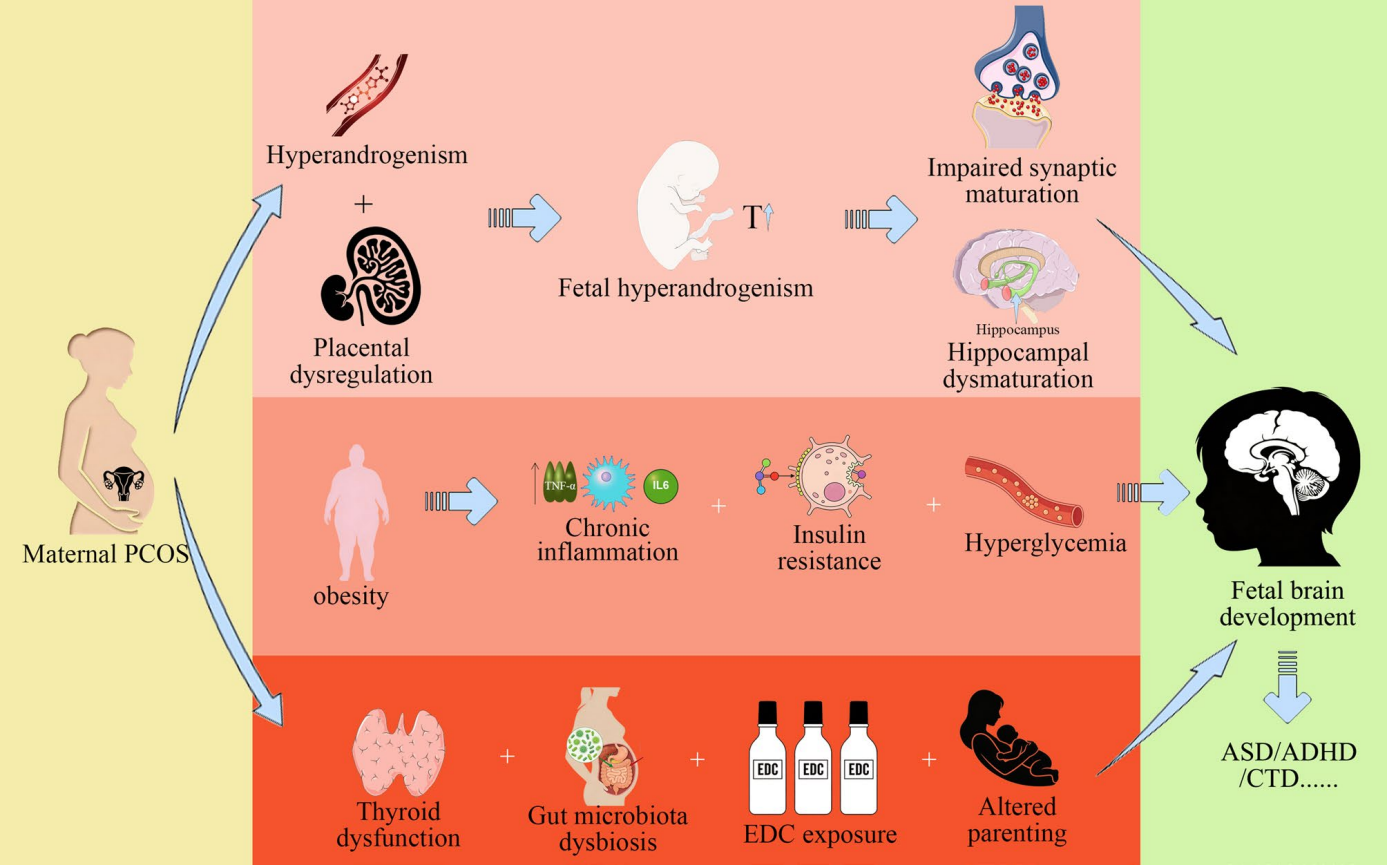
Potential pathways linking maternal polycystic ovary syndrome (PCOS) to neuropsychiatric disorders (NPDs) in offspring. PCOS, polycystic ovarian syndrome; EDC, endocrine-disrupting chemical.

Beyond the established roles mentioned above, several emerging mechanisms highlighted in Kahn’s review may further explain the elevated neuropsychiatric risks in offspring of mothers with PCOS including placental inflammation, systemic oxidative stress, maternal gut microbiota dysbiosis, and exposure to environmental endocrine-disrupting chemicals (EDCs) [79]. Furthermore, psychosocial pathways—such as altered parenting behaviors resulting from maternal psychiatric comorbidities—may act as important modifiers of offspring neurohealth [79]. However, the studies currently included in this meta-analysis did not provide specific discussions or granular data regarding these mechanisms. Therefore, further research specifically investigating these mechanisms in the context of PCOS is warranted to elucidate their relative contributions to offspring neurodevelopmental outcomes. We visually summarized the complex interplay of these established and emerging pathways in Fig. 2.

There are several strength and limitations in our study. Firstly, this review included studies published in English, which may lead to language bias, as English studies are more likely to report positive results [84]. Secondly, since this is a meta-analysis based on pooled data, we were unable to control for confounding factors in our analysis and could only use adjusted OR and HR as much as possible. Thirdly, due to data limitations, we were unable to perform subgroup analyses for all results and we were unable to perform subgroup analyses based on PCOS categories. Eventually, the reliance on self-reported PCOS diagnosis in some included studies may introduce potential misclassification bias.

## Conclusion

In conclusion, this large-scale meta-analysis identifies associations between maternal PCOS and increased odds of a range of neuropsychiatric disorders in offspring. These findings suggest that children of mothers with PCOS may benefit from developmental monitoring, although causal relationships remain to be established. Future research should aim to confirm these associations and investigate underlying mechanisms. 

## Supplementary Information

Below is the link to the electronic supplementary material.

Supplementary Material 1: Supplemental figure 1. Funnel plots of individual study results (unadjusted). ASD autism spectrum disorder, ADHD attention deficit hyperactivity disorder, CTD chronic tic disorder, ASQ Ages and Stages Questionnaire

Supplementary Material 2: Supplemental figure 2. Forest plots of maternal PCOS on the neurodevelopment in offspring (adjusted). PCOS polycystic ovary syndrome, ASD autism spectrum disorder, ADHD attention deficit hyperactivity disorder, CTD chronic tic disorder, ASQ Ages and Stages Questionnaire

Supplementary Material 3: Supplemental figure 3. Forest plots of maternal PCOS on the neurodevelopment in offspring (unadjusted). PCOS polycystic ovary syndrome, ASD autism spectrum disorder, ADHD attention deficit hyperactivity disorder, CTD chronic tic disorder, ASQ Ages and Stages Questionnaire

Supplementary Material 4: Supplemental figure 4. Funnel plots of individual study results (adjusted). ASD autism spectrum disorder, ADHD attention deficit hyperactivity disorder, CTD chronic tic disorder, ASQ Ages and Stages Questionnaire

Supplementary Material 5: Supplemental figure 5. Forest plot showing the impact of maternal polycystic ovary syndrome (PCOS) on ASQ subscale scores in offspring

Supplementary Material 6

Supplementary Material 7

Supplementary Material 8

Supplementary Material 9

## Data Availability

The data underlying this article will be shared on reasonable request to the corresponding author.

## Abbreviations

PCOS: Polycystic ovary syndrome
ASD: Autism spectrum disorder
ADHD: Attention deficit hyperactivity disorder
CTD: Chronic tic disorder
NPD: Neuropsychiatric disorders
