# Supplementary figures and images for: Neuropsychiatric disorders in children of mothers with polycystic ovary syndrome: a systematic review and meta-analysis
Source: BMC Psychiatry. 2026 Apr 4;26:411. doi: 10.1186/s12888-026-08047-4 (PMC13188356; doi:10.1186/s12888-026-08047-4)

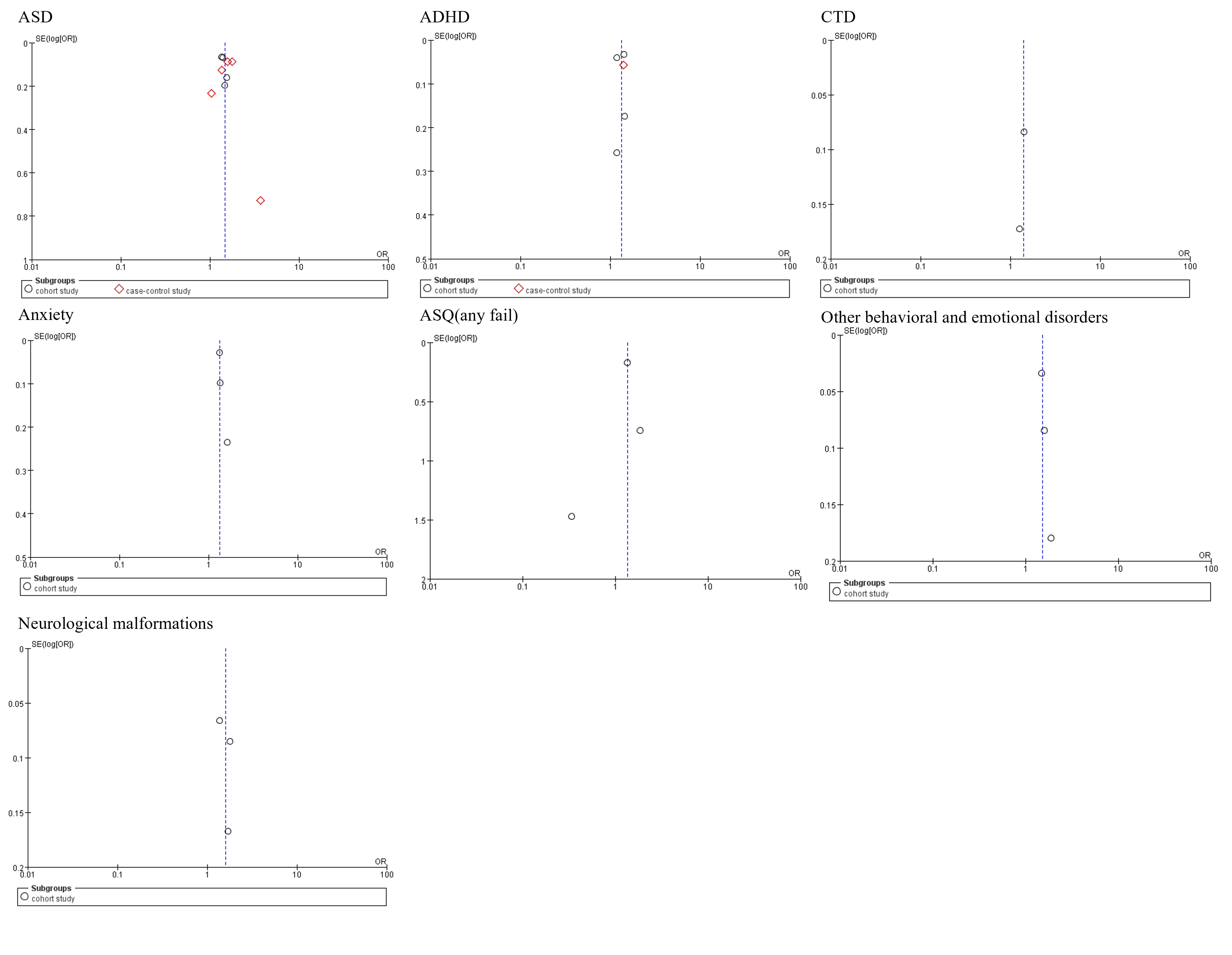

Supplement: Supplementary file 1 — Supplementary Material 1: Supplemental figure 1. Funnel plots of individual study results (unadjusted). ASD autism spectrum disorder, ADHD attention deficit hyperactivity disorder, CTD chronic tic disorder, ASQ Ages and Stages Questionnaire [file 12888_2026_8047_MOESM1_ESM.png]

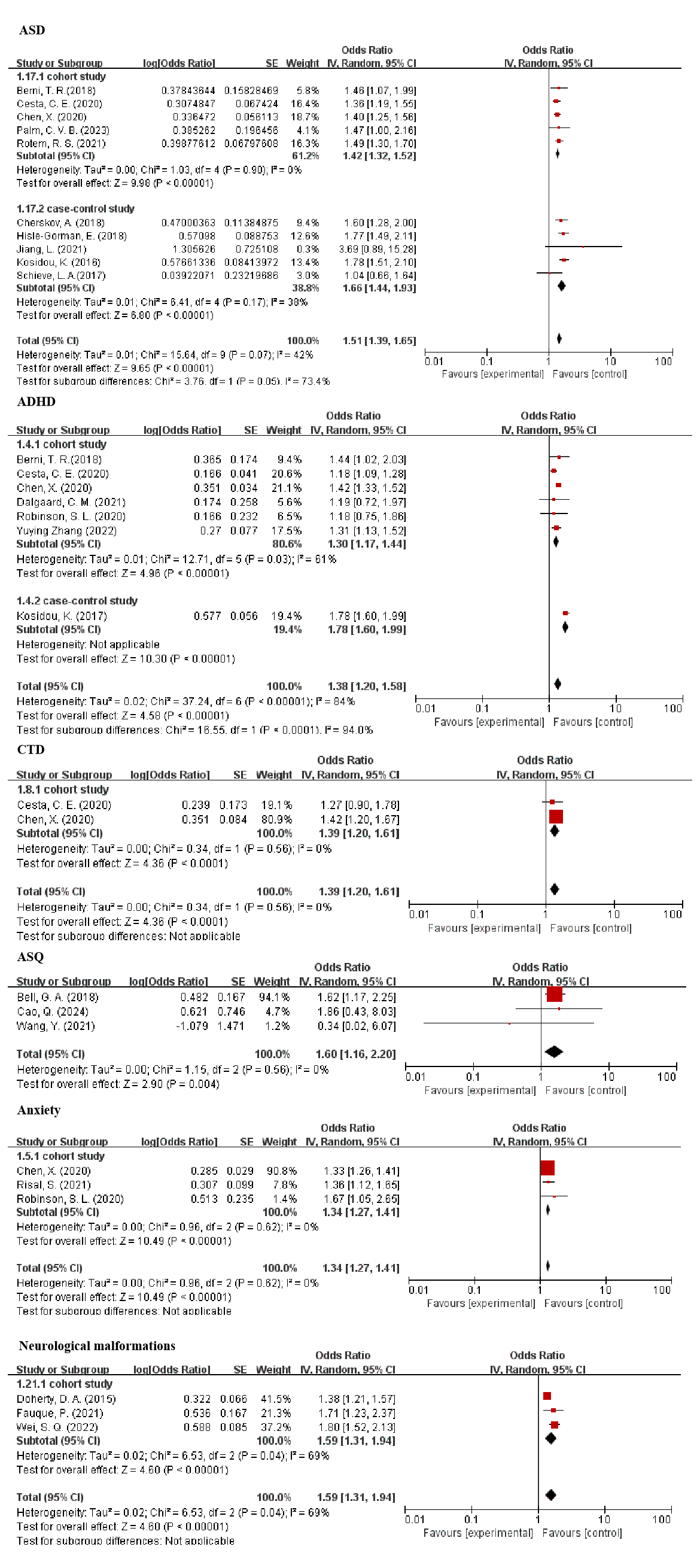

Supplement: Supplementary file 2 — Supplementary Material 2: Supplemental figure 2. Forest plots of maternal PCOS on the neurodevelopment in offspring (adjusted). PCOS polycystic ovary syndrome, ASD autism spectrum disorder, ADHD attention deficit hyperactivity disorder, CTD chronic tic disorder, ASQ Ages and Stages Questionnaire [file 12888_2026_8047_MOESM2_ESM.png]

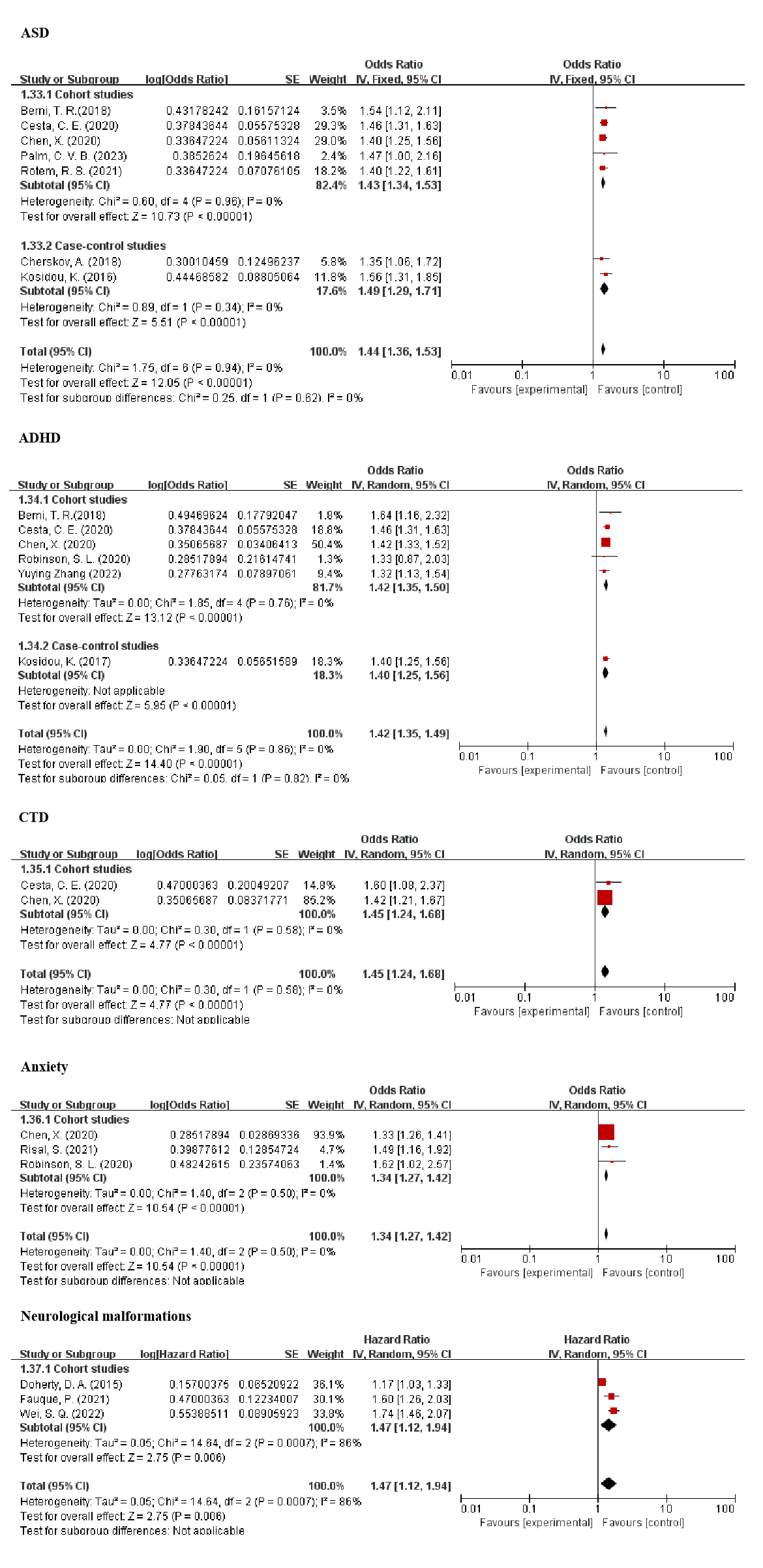

Supplement: Supplementary file 3 — Supplementary Material 3: Supplemental figure 3. Forest plots of maternal PCOS on the neurodevelopment in offspring (unadjusted). PCOS polycystic ovary syndrome, ASD autism spectrum disorder, ADHD attention deficit hyperactivity disorder, CTD chronic tic disorder, ASQ Ages and Stages Questionnaire [file 12888_2026_8047_MOESM3_ESM.png]

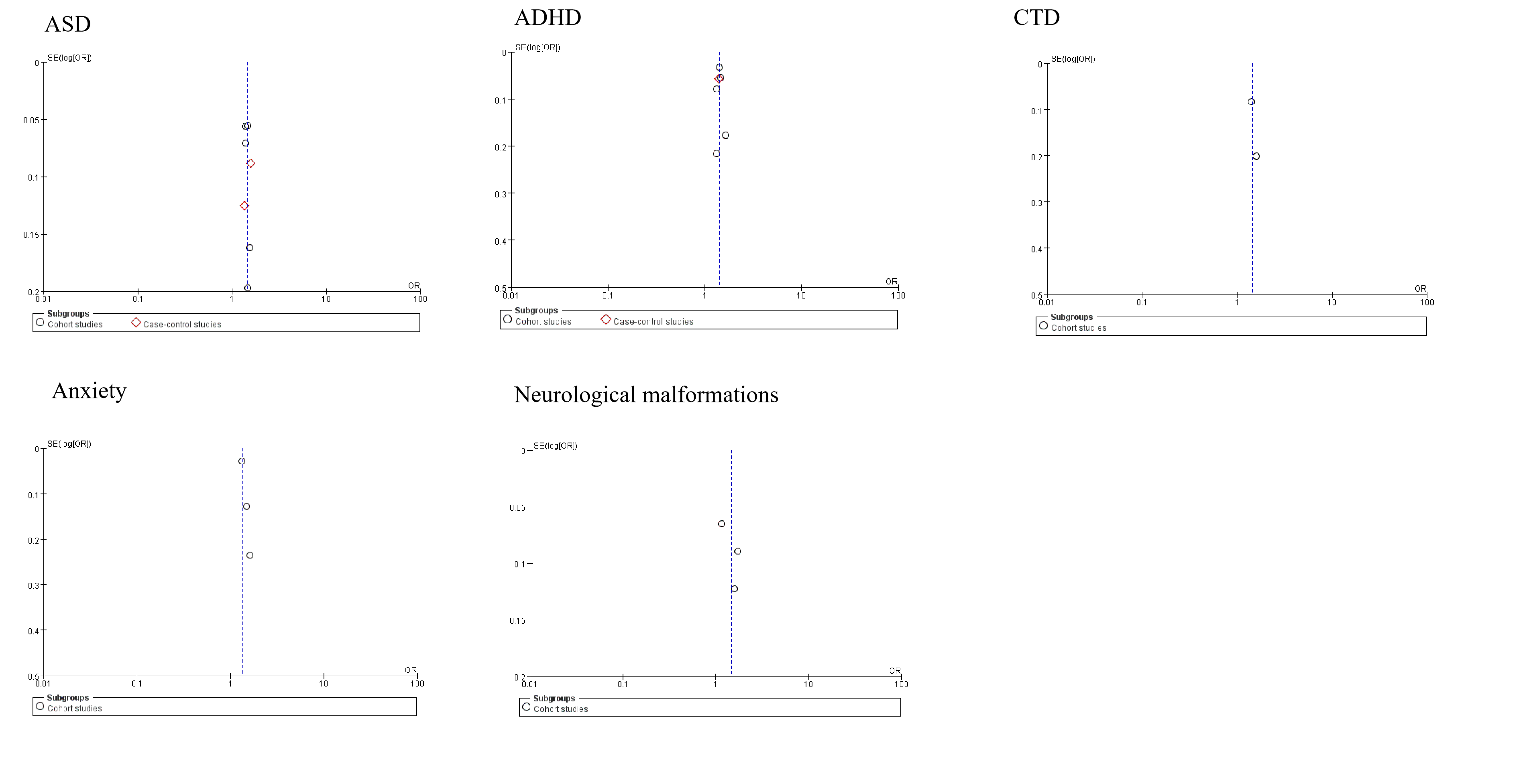

Supplement: Supplementary file 4 — Supplementary Material 4: Supplemental figure 4. Funnel plots of individual study results (adjusted). ASD autism spectrum disorder, ADHD attention deficit hyperactivity disorder, CTD chronic tic disorder, ASQ Ages and Stages Questionnaire [file 12888_2026_8047_MOESM4_ESM.png]

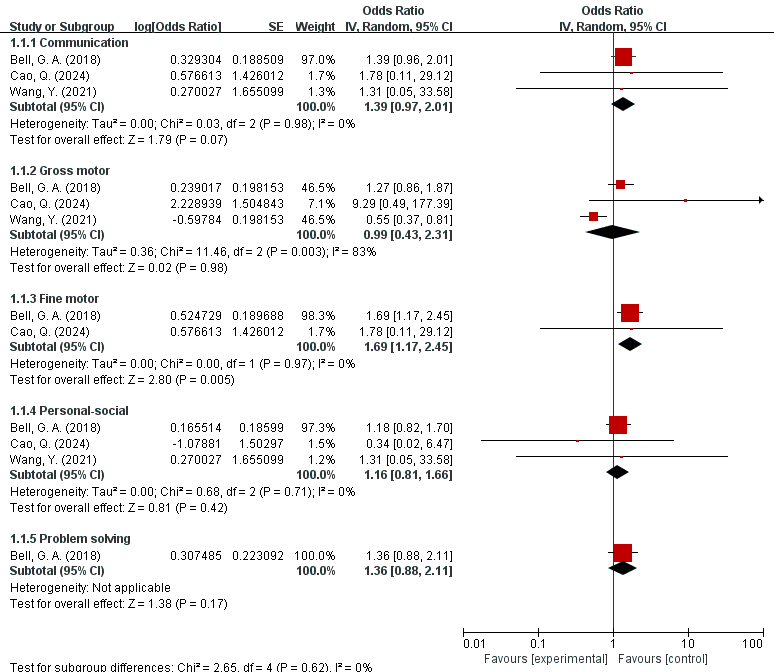

Supplement: Supplementary file 5 — Supplementary Material 5: Supplemental figure 5. Forest plot showing the impact of maternal polycystic ovary syndrome (PCOS) on ASQ subscale scores in offspring [file 12888_2026_8047_MOESM5_ESM.png]
