# Supplementary material for: Neuropsychiatric disorders in children of mothers with polycystic ovary syndrome: a systematic review and meta-analysis
Source: BMC Psychiatry. 2026 Apr 4;26:411. doi: 10.1186/s12888-026-08047-4 (PMC13188356; doi:10.1186/s12888-026-08047-4)
Supplement: Supplementary file 6 — Supplementary Material 6 [file 12888_2026_8047_MOESM6_ESM.docx]

Table S1. Search strategy.

| **Pubmed** |
| --- |
| #1 "Polycystic Ovary Syndrome"[Mesh] OR "Polycystic Ovary Syndrome" OR "PCOS" OR "polycystic ovarian syndrome" OR "Stein-Leventhal syndrome" |
| #2 "neurodevelop*"[tiab] OR "mental health"[Mesh] OR "cognition"[Mesh] OR "cognitive dysfunction"[Mesh] OR "behavior"[Mesh] OR "child behavior"[Mesh] OR "attention deficit disorder"[tiab] OR "autism spectrum disorder"[Mesh] OR "depression"[Mesh] OR "anxiety"[Mesh] OR "intelligence"[Mesh] OR "executive function"[Mesh] OR "developmental disabilities"[Mesh] OR "Neurologic Diseases"[tiab] OR "Neurodevelopmental Disorders"[Mesh] OR "Epilepsy"[Mesh] OR "Cerebral Palsy"[Mesh] OR "Brain Injuries"[Mesh] OR "Neuromuscular Diseases"[Mesh] OR "Seizures"[Mesh] OR "Movement Disorders"[Mesh] OR "Motor Disorders"[Mesh] OR "neurodegenerative disease*"[tiab] OR "neurological disorder*"[tiab] OR "brain disorder*"[tiab] OR "nervous system disorder*"[tiab] OR "cerebral disorder*"[tiab] OR "developmental delay*"[tiab] OR “schizophrenia”[tiab] OR “bipolar disorder” [tiab] OR “eating disorder” [tiab] OR “motor*” [tiab] OR  ("Gesell Developmental Schedules"[Title/Abstract]) OR  ("Vineland Adaptive Behavior Scales"[Title/Abstract]) OR  ("Bayley Scales of Infant Development"[Title/Abstract] OR BSID[Title/Abstract]) OR  ("Denver Developmental Screening Test"[Title/Abstract] OR DDST[Title/Abstract]) OR  ("Griffiths Mental Development Scales"[Title/Abstract]) OR  ("Ages and Stages Questionnaire"[Title/Abstract] OR ASQ[Title/Abstract]) OR  ("Child Development Assessment"[Title/Abstract] OR CDA[Title/Abstract]) OR  ("Developmental Coordination Disorder Questionnaire"[Title/Abstract] OR DCDQ[Title/Abstract]) OR  ("Neonatal Behavioral Assessment Scale"[Title/Abstract] OR NBAS[Title/Abstract]) OR  ("Neuropsychological Developmental Scale for Children 0-6 Years"[Title/Abstract] OR "Erxin Scale"[Title/Abstract]) OR  ("China Developmental Scale for Children"[Title/Abstract] OR CDCC[Title/Abstract]) OR  ("Wechsler Intelligence Scale for Children"[Title/Abstract] OR WISC[Title/Abstract]) OR  ("Wechsler Preschool and Primary Scale of Intelligence"[Title/Abstract] OR WPPSI[Title/Abstract]) OR  ("Stanford-Binet Intelligence Scales"[Title/Abstract]) OR  ("McCarthy Scales of Children's Abilities"[Title/Abstract] OR MSCA[Title/Abstract]) OR  ("Kaufman Assessment Battery for Children"[Title/Abstract] OR K-ABC[Title/Abstract]) OR  ("Raven's Progressive Matrices"[Title/Abstract] OR RPM[Title/Abstract]) OR  ("Differential Ability Scales"[Title/Abstract] OR DAS[Title/Abstract]) OR  ("Test of Nonverbal Intelligence"[Title/Abstract] OR TONI[Title/Abstract]) OR  ("Cognitive Assessment System"[Title/Abstract] OR CAS[Title/Abstract]) OR  ("Reynolds Intellectual Assessment Scales"[Title/Abstract] OR RIAS[Title/Abstract]) OR  ("Early Language Milestones Scale"[Title/Abstract] OR ELM[Title/Abstract]) OR  ("Peabody Picture Vocabulary Test"[Title/Abstract] OR PPVT[Title/Abstract]) OR  ("Language Development Scale for Children"[Title/Abstract] OR LDSC[Title/Abstract]) OR  ("Clinical Evaluation of Language Fundamentals"[Title/Abstract] OR CELF[Title/Abstract]) OR  ("Preschool Language Scale"[Title/Abstract] OR PLS[Title/Abstract]) OR  ("Verbal Behavior Milestones Assessment and Placement Program"[Title/Abstract] OR VB-MAPP[Title/Abstract]) OR  ("Chinese Communicative Development Inventory"[Title/Abstract] OR CCDI[Title/Abstract]) OR  ("Expressive Vocabulary Test"[Title/Abstract] OR EVT[Title/Abstract]) OR  ("Gross Motor Function Measure"[Title/Abstract] OR GMFM[Title/Abstract]) OR  ("Quality of Upper Extremity Skills Test"[Title/Abstract] OR QUEST[Title/Abstract]) OR  ("Sensory Integration and Praxis Tests"[Title/Abstract] OR SIPT[Title/Abstract]) OR  ("Movement Assessment Battery for Children"[Title/Abstract] OR MABC[Title/Abstract]) OR  ("Alberta Infant Motor Scale"[Title/Abstract] OR AIMS[Title/Abstract]) OR  ("Test of Infant Motor Performance"[Title/Abstract] OR TIMP[Title/Abstract]) OR  ("Child Behavior Checklist"[Title/Abstract] OR CBCL[Title/Abstract]) OR  ("Strengths and Difficulties Questionnaire"[Title/Abstract] OR SDQ[Title/Abstract]) OR  ("Social Communication Questionnaire"[Title/Abstract] OR SCQ[Title/Abstract]) OR  ("Autism Behavior Checklist"[Title/Abstract] OR ABC[Title/Abstract]) OR  ("Conners Rating Scales"[Title/Abstract] OR Conners[Title/Abstract]) OR  ("Social Responsiveness Scale"[Title/Abstract] OR SRS[Title/Abstract]) OR  ("Screen for Child Anxiety Related Emotional Disorders"[Title/Abstract] OR SCARED[Title/Abstract]) OR  ("Children's Depression Inventory"[Title/Abstract] OR CDI[Title/Abstract]) OR  ("Infant-Toddler Social and Emotional Assessment"[Title/Abstract] OR ITSEA[Title/Abstract]) OR  ("Autism Diagnostic Observation Schedule"[Title/Abstract] OR ADOS[Title/Abstract]) OR  ("Autism Diagnostic Interview-Revised"[Title/Abstract] OR ADI-R[Title/Abstract]) OR  ("Modified Checklist for Autism in Toddlers"[Title/Abstract] OR M-CHAT[Title/Abstract]) OR  ("Childhood Autism Rating Scale"[Title/Abstract] OR CARS[Title/Abstract]) OR  ("Autism Treatment Evaluation Checklist"[Title/Abstract] OR ATEC[Title/Abstract]) OR  ("Vanderbilt ADHD Diagnostic Rating Scale"[Title/Abstract]) OR  ("ADHD Rating Scale"[Title/Abstract] OR ADHD-RS[Title/Abstract]) OR  ("Swanson, Nolan, and Pelham Rating Scale"[Title/Abstract] OR SNAP-IV[Title/Abstract]) OR  ("Behavior Rating Inventory of Executive Function"[Title/Abstract] OR BRIEF[Title/Abstract]) OR  ("Child PTSD Symptom Scale"[Title/Abstract] OR CPSS[Title/Abstract]) OR  ("Children's Sleep Habits Questionnaire"[Title/Abstract] OR CSHQ[Title/Abstract]) OR  ("Children's Eating Behavior Questionnaire"[Title/Abstract] OR CEBQ[Title/Abstract]) OR  ("Childhood Trauma Questionnaire"[Title/Abstract] OR CTQ[Title/Abstract]) OR  ("Neonatal Behavioral Assessment Scale"[Title/Abstract] OR NBAS[Title/Abstract] OR "Brazelton Scale"[Title/Abstract]) OR  ("Enjoji Infant Analytical Developmental Test"[Title/Abstract] OR "Enjoji Scale"[Title/Abstract]) |
| #3 ("offspring"[Title/Abstract] OR "child"[Mesh] OR "infant"[Mesh] OR "fetus"[Mesh] OR "prenatal exposure delayed effects"[Mesh] OR "infant"[Title/Abstract] OR "fetus"[Title/Abstract] OR "prenatal exposure delayed effects"[Title/Abstract] OR "children"[Title/Abstract] OR “adolescent” [Title/Abstract]) |
| #1 AND #2 AND #3 |
| **Embase**: |
| 1. exp polycystic ovary syndrome/ OR (PCOS OR polycystic ovarian syndrome OR "Stein-Leventhal syndrome").ti,ab,kw |
| 2. exp mental health/ or exp cognition/ or exp cognitive defect/ or exp behavior/ or exp child behavior/ or exp autism/ or exp depression/ or exp anxiety/ or exp intelligence/ or exp executive function/ or exp developmental disorder/ or exp neurodevelopmental disorder/ or exp epilepsy/ or exp cerebral palsy/ or exp brain injury/ or exp neuromuscular disease/ or exp seizure/ or exp movement disorder/ or exp motor disorder/ or exp neurodegenerative disease/ or exp nervous system disease/ or (neurodevelop* or "attention deficit hyperactivity disorder" or "neurologic disease" or "brain disorder*" or "nervous system disorder*" or "cerebral disorder*" or "developmental delay*" or “schizophrenia” or “bipolar disorder” or “eating disorder” or ("Gesell Developmental Schedules") OR  ("Vineland Adaptive Behavior Scales") OR  ("Bayley Scales of Infant Development" OR BSID) OR  ("Denver Developmental Screening Test" OR DDST) OR  ("Griffiths Mental Development Scales") OR  ("Ages and Stages Questionnaire" OR ASQ) OR  ("Child Development Assessment" OR CDA) OR  ("Developmental Coordination Disorder Questionnaire" OR DCDQ) OR  ("Neonatal Behavioral Assessment Scale" OR NBAS) OR  ("Neuropsychological Developmental Scale for Children 0-6 Years" OR "Erxin Scale") OR  ("China Developmental Scale for Children" OR CDCC) OR  ("Wechsler Intelligence Scale for Children" OR WISC) OR  ("Wechsler Preschool and Primary Scale of Intelligence" OR WPPSI) OR  ("Stanford-Binet Intelligence Scales") OR  ("McCarthy Scales of Children's Abilities" OR MSCA) OR  ("Kaufman Assessment Battery for Children" OR K-ABC) OR  ("Raven's Progressive Matrices" OR RPM) OR  ("Differential Ability Scales" OR DAS) OR  ("Test of Nonverbal Intelligence" OR TONI) OR  ("Cognitive Assessment System" OR CAS) OR  ("Reynolds Intellectual Assessment Scales" OR RIAS) OR  ("Early Language Milestones Scale" OR ELM) OR  ("Peabody Picture Vocabulary Test" OR PPVT) OR  ("Language Development Scale for Children" OR LDSC) OR  ("Clinical Evaluation of Language Fundamentals" OR CELF) OR  ("Preschool Language Scale" OR PLS) OR  ("Verbal Behavior Milestones Assessment and Placement Program" OR VB-MAPP) OR  ("Chinese Communicative Development Inventory" OR CCDI) OR  ("Expressive Vocabulary Test" OR EVT) OR  ("Gross Motor Function Measure" OR GMFM) OR  ("Quality of Upper Extremity Skills Test" OR QUEST) OR  ("Sensory Integration and Praxis Tests" OR SIPT) OR  ("Movement Assessment Battery for Children" OR MABC) OR  ("Alberta Infant Motor Scale" OR AIMS) OR  ("Test of Infant Motor Performance" OR TIMP) OR  ("Child Behavior Checklist" OR CBCL) OR  ("Strengths and Difficulties Questionnaire" OR SDQ) OR  ("Social Communication Questionnaire" OR SCQ) OR  ("Autism Behavior Checklist" OR ABC) OR  ("Conners Rating Scales" OR Conners) OR  ("Social Responsiveness Scale" OR SRS) OR  ("Screen for Child Anxiety Related Emotional Disorders" OR SCARED) OR  ("Children's Depression Inventory" OR CDI) OR  ("Infant-Toddler Social and Emotional Assessment" OR ITSEA) OR  ("Autism Diagnostic Observation Schedule" OR ADOS) OR  ("Autism Diagnostic Interview-Revised" OR ADI-R) OR  ("Modified Checklist for Autism in Toddlers" OR M-CHAT) OR  ("Childhood Autism Rating Scale" OR CARS) OR  ("Autism Treatment Evaluation Checklist" OR ATEC) OR  ("Vanderbilt ADHD Diagnostic Rating Scale") OR  ("ADHD Rating Scale" OR ADHD-RS) OR  ("Swanson, Nolan, and Pelham Rating Scale" OR SNAP-IV) OR  ("Behavior Rating Inventory of Executive Function" OR BRIEF) OR  ("Child PTSD Symptom Scale" OR CPSS) OR  ("Children's Sleep Habits Questionnaire" OR CSHQ) OR  ("Children's Eating Behavior Questionnaire" OR CEBQ) OR  ("Childhood Trauma Questionnaire" OR CTQ) OR  ("Neonatal Behavioral Assessment Scale" OR NBAS OR "Brazelton Scale") OR  ("Enjoji Infant Analytical Developmental Test" OR "Enjoji Scale")).ti,ab,kw. |
| 3. exp offspring/ OR exp child/ OR children OR exp infant/ OR exp fetus/ OR exp prenatal exposure/ OR exp adolescent/ |
| 1 AND 2 AND 3 |
| **Cochrane** |
| 1. "Polycystic Ovary Syndrome"[Mesh] OR (Polycystic Ovary Syndrome OR PCOS OR polycystic ovarian syndrome OR Stein-Leventhal syndrome):ti,ab,kw |
| 1. (neurodevelop* OR Mental Health OR Mental disease OR Cognition OR Cognitive Dysfunction OR Behavior OR attention deficit disorder OR ADHD OR Autism OR Depression OR Anxiety OR Intelligence OR Executive Function OR Developmental Disabilities OR Neurologic* Diseases OR developmental Disorders OR Epilepsy OR Cerebral Palsy OR Brain Injuries OR Neuromuscular Diseases OR Seizures OR Movement Disorders OR Motor* OR neurodegenerative disease* OR neurological disorder* OR brain disorder* OR nervous system disorder* OR cerebral disorder* OR developmental delay* OR schizophrenia OR bipolar disorder OR eating disorder OR (Gesell Developmental Schedules) OR   ("Bayley Scales of Infant Development" OR BSID) OR  ("Denver Developmental Screening Test" OR DDST) OR  "Griffiths Mental Development Scales" OR  ("Ages and Stages Questionnaire" OR ASQ) OR  ("Child Development Assessment" OR CDA) OR  ("Developmental Coordination Disorder Questionnaire" OR DCDQ) OR  ("Neonatal Behavioral Assessment Scale" OR NBAS OR "Brazelton Scale") OR  ("Neuropsychological Developmental Scale for Children 0-6 Years" OR "Erxin Scale") OR  ("China Developmental Scale for Children" OR CDCC) OR  ("Wechsler Intelligence Scale for Children" OR WISC) OR  ("Wechsler Preschool and Primary Scale of Intelligence" OR WPPSI) OR  "Stanford-Binet Intelligence Scales" OR  ("McCarthy Scales of Children's Abilities" OR MSCA) OR  ("Kaufman Assessment Battery for Children" OR K-ABC) OR  ("Raven's Progressive Matrices" OR RPM) OR  ("Differential Ability Scales" OR DAS) OR  ("Test of Nonverbal Intelligence" OR TONI) OR  ("Cognitive Assessment System" OR CAS) OR  ("Reynolds Intellectual Assessment Scales" OR RIAS) OR  ("Early Language Milestones Scale" OR ELM) OR  ("Peabody Picture Vocabulary Test" OR PPVT) OR  ("Language Development Scale for Children" OR LDSC) OR  ("Clinical Evaluation of Language Fundamentals" OR CELF) OR  ("Preschool Language Scale" OR PLS) OR  ("Verbal Behavior Milestones Assessment and Placement Program" OR VB-MAPP) OR  ("Chinese Communicative Development Inventory" OR CCDI) OR  ("Expressive Vocabulary Test" OR EVT) OR  ("Gross Motor Function Measure" OR GMFM) OR  ("Quality of Upper Extremity Skills Test" OR QUEST) OR  ("Sensory Integration and Praxis Tests" OR SIPT) OR  ("Movement Assessment Battery for Children" OR MABC) OR  ("Alberta Infant Motor Scale" OR AIMS) OR  ("Test of Infant Motor Performance" OR TIMP) OR  ("Child Behavior Checklist" OR CBCL) OR  ("Strengths and Difficulties Questionnaire" OR SDQ) OR  ("Social Communication Questionnaire" OR SCQ) OR  ("Autism Behavior Checklist" OR ABC) OR  ("Conners Rating Scales" OR Conners) OR  ("Social Responsiveness Scale" OR SRS) OR  ("Screen for Child Anxiety Related Emotional Disorders" OR SCARED) OR  ("Children's Depression Inventory" OR CDI) OR  ("Infant-Toddler Social and Emotional Assessment" OR ITSEA) OR  ("Autism Diagnostic Observation Schedule" OR ADOS) OR  ("Autism Diagnostic Interview-Revised" OR ADI-R) OR  ("Modified Checklist for Autism in Toddlers" OR M-CHAT) OR  ("Childhood Autism Rating Scale" OR CARS) OR  ("Autism Treatment Evaluation Checklist" OR ATEC) OR  "Vanderbilt ADHD Diagnostic Rating Scale" OR  ("ADHD Rating Scale" OR ADHD-RS) OR  ("Swanson, Nolan, and Pelham Rating Scale" OR SNAP-IV) OR  ("Behavior Rating Inventory of Executive Function" OR BRIEF) OR  ("Child PTSD Symptom Scale" OR CPSS) OR  ("Children's Sleep Habits Questionnaire" OR CSHQ) OR  ("Children's Eating Behavior Questionnaire" OR CEBQ) OR  ("Childhood Trauma Questionnaire" OR CTQ) OR  ("Enjoji Infant Analytical Developmental Test" OR "Enjoji Scale")):ti,ab,kw |
| 1. (offspring OR child OR children OR infant OR fetus OR prenatal exposure OR adolescent):ti,ab,kw |
| 1 AND 2 AND 3 |
