# Supplementary material for: Neuropsychiatric disorders in children of mothers with polycystic ovary syndrome: a systematic review and meta-analysis
Source: BMC Psychiatry. 2026 Apr 4;26:411. doi: 10.1186/s12888-026-08047-4 (PMC13188356; doi:10.1186/s12888-026-08047-4)
Supplement: Supplementary file 7 — Supplementary Material 7 [file 12888_2026_8047_MOESM7_ESM.docx]

Table S2. Quality assessment of included studies in meta-analysis using Newcastle–Ottawa Scale.

|  | **Selection** | | | | **Comparability** | **Exposure/Outcome** | | | **Total** | **AHRQ** |
| --- | --- | --- | --- | --- | --- | --- | --- | --- | --- | --- |
|  | q1 | q2 | q3 | q4 | q1 | q1 | q2 | q3 |  |  |
| **Cohort study** |  |  |  |  |  |  |  |  |  |  |
| Bell, G. A. (2018) | * | * |  | * | * | * | * | * | 7 | Good |
| Berni, T. R.(2018) | * | * | * |  | * | * |  | * | 6 | Good |
| Cao, Q. (2024) | * | * | * | * | * | * | * | * | 8 | Good |
| Cesta, C. E. (2020) | * | * | * |  | * | * |  | * | 6 | Good |
| Chen, X. (2020) | * | * | * |  | * | * | * | * | 7 | Good |
| Dalgaard, C. M. (2021) | * | * | * | * | * |  | * | * | 7 | Good |
| Doherty, D. A. (2015) | * | * | * | * | * | * | * | * | 8 | Good |
| Fauque, P. (2021) | * | * | * | * | * | * | * |  | 7 | Good |
| Palm, C. V. B. (2023) | * | * | * |  | * | * | * | * | 7 | Good |
| Risal, S. (2021) | * | * | * | * | * | * |  | * | 7 | Good |
| Robinson, S. L. (2020) | * | * |  | * | * | * |  | * | 6 | Good |
| Rotem, R. S. (2021) | * | * |  | * | * | * | * | * | 8 | Good |
| Wei, S. Q. (2022) | * | * |  | * | * | * | * | * | 8 | Good |
| Yuying Zhang (2022) | * | * |  | * | * |  | * | * | 6 | Good |
| **Case-control study** |  |  |  |  |  |  |  |  |  |  |
| Cherskov, A. (2018) | * | * | * | * | * | * | * |  | 7 | Good |
| Hisle-Gorman, E. (2018) | * | * |  | * | * | * | * |  | 6 | Good |
| Jiang, L. (2021) | * | * |  | * | * | * | * |  | 6 | Good |
| Kosidou, K. (2016) | * | * | * | * | * | * | * | * | 8 | Good |
| Kosidou, K. (2017) | * | * | * | * | * | * | * | * | 8 | Good |
| Wang, Y. (2021) | * |  |  | * | * | * | * |  | 5 | Fair |
| Schieve, L. A.(2017) | * | * | * | * | * | * | * |  | 7 | Good |

**Abbreviations:** AHQR, agency for healthcare research and quality. q1-q4: questions.

Quality of a cross-sectional study was also assessed using cohort study criteria of Newcastle–Ottawa Scale;
