# Supplementary material for: Neuropsychiatric disorders in children of mothers with polycystic ovary syndrome: a systematic review and meta-analysis
Source: BMC Psychiatry. 2026 Apr 4;26:411. doi: 10.1186/s12888-026-08047-4 (PMC13188356; doi:10.1186/s12888-026-08047-4)
Supplement: Supplementary file 8 — Supplementary Material 8 [file 12888_2026_8047_MOESM8_ESM.docx]

Table S3. The adjusted variables of adjusted effect size.

| Author(year) | Outcomes | Adjusted variables | Maternal age | Family history of neuropsychiatric disorders | prenatal exposures (smoking/alcohol ) | maternal body mass index |
| --- | --- | --- | --- | --- | --- | --- |
| Bell, G. A. (2018) | ASQ | Maternal age, body mass index, race/ethnicity, marital status, private insurance, education, smoking during pregnancy, drinking during pregnancy, any diabetes and plurality. | √ | × | √ | √ |
| Berni T. R.(2018) | ASD, ADHD | Age (62 years), body mass index (BMI) category (,25 kg/m2, 25 to 30 kg/m2, .30 kg/m2), and primary-care practice. | √ | × | × | √ |
| Cesta C. E. (2020) | ASD, ADHD, CTD | Offspring sex and year of birth, maternal age at child’s birth, maternal education, maternal region of birth, and maternal and paternal lifetime history of psychiatric disorders. | √ | √ | × | × |
| Chen X. (2020) | ASD, ADHD, CTD, Anxiety, Other  behavioral and emotional disorders (mood disorders, eating disorders, Sleeping disorders, personality disorders, intellectual disabilities, specific  developmental disorders) | Maternal age, mother’s country of birth (Finland or not), mother married at birth (yes/no), maternal smoking (yes/no), parity (0 or 1), maternal psychiatric disorder (yes/no), maternal purchase of N05 and N06 during pregnancy (yes/no) and maternal systemic inflammatory disease (yes/no). | √ | √ | √ | × |
| Cherskov, A. (2018) | ASD | Maternal age (at childbirth), depression, anxiety, schizophrenia-related illness, complications at childbirth, obesity, diabetes, and gestational diabetes | √ | √ | × | √ |
| Doherty, D. A. (2015) | Neurological malformations | In vitro fertilization, ethnicity (Caucasian, Indigenous, Asian, other), multiple pregnancy, maternal age (younger than 20, 20–29, 30–39, 40 years or older), parity (zero, one to four, five or more), time epoch of birth (years less than 1990, 1990–1999, 2000–2011), smoking during pregnancy (yes, no, unreported), maternal hypertension, asthma, cardiovascular conditions, pre-existing and gestational diabetes, preeclampsia, male gender, gestational age at delivery (less than 32, 33–34, 35–36, 37–40, 41 weeks or greater), major congenital anomalies, neonatal admission to special care nursery after birth. Adjustment for being born small for gestational age was made for the outcomes endocrine, nutritional and metabolic, metabolic, psychological development, nervous system, eye and adnexa, ear and mastoid process, circulatory system; upper and lower and other respiratory tract infections and asthma, digestive system, and musculoskeletal and connective tissue disorders. | √ | × | √ | × |
| Fauque, P. (2021) | Neurological malformations | age, primiparity, smoking, obesity, history of high blood pressure or diabetes, new-born sex and mode of conception. | √ | × | √ | × |
| Kosidou(2016) | ASD | Maternal age, paternal age, parental psychiatric history, household income, parental education and mother’s country of birth，obstetric complications (gestational diabetes, Apgar score at 5min, gestational age, size for gestational age and pre-eclampsia) | √ | √ | × | × |
| Kosidou K. (2017) | ADHD | Maternal age, paternal age, birth order, parental psychiatric history, household income, parental education, and mother’s country of birth, obstetric complications (Apgar score at 5 minutes, preterm birth, small for gestational  age, and preeclampsia) | √ | √ | × | × |
| Risal, S. (2021) | Anxiety | Highest attained maternal education level (primary and secondary education, upper secondary education, postsecondary/post-graduate education), Maternal and paternal lifetime history of psychiatric disorders, Region of birth (Nordic/non-Nordic), child’s sex and year of birth , maternal body mass index (BMI) | × | √ | × | √ |
| Robinson S. L. (2020) | ADHD | Maternal age, race, education, insurance status, marital status, smoking, and father’s age difference, maternal and paternal history of affective disorder and maternal body mass index, child’s sex. | √ | √ | √ | √ |
| Rotem R. S. (2021) | ASD | Calendar year, maternal age, residential district, socioeconomic status, and high minority (Israeli Arabs and/or Jewish Orthodox) and immigrant subpopulations at 1residential enumeration area，maternal psychiatric morbidity | √ | √ | × | × |
| Wei, S. Q. (2022) | Neurological malformations | Maternal age, parity, ART, maternal comorbidity, substance use disorder, socioeconomic deprivation and year of birth. | √ | × | × | × |
| Yuying Zhang (2022) | ADHD | Maternal age at childbirth, maternal education, marital status, household income, passive smoking during pregnancy, pre-pregnant body mass index; paternal age at childbirth and paternal education; child sex and child age at ADHD assessment. | √ | × | √ | √ |

OR odds ratio, HR hazard ratio, ASD autism spectrum disorder, ADHD attention deficit hyperactivity disorder, CTD chronic tic disorder, ASQ Ages & Stages Questionnaires.
