# Supplementary material for: Neuropsychiatric disorders in children of mothers with polycystic ovary syndrome: a systematic review and meta-analysis
Source: BMC Psychiatry. 2026 Apr 4;26:411. doi: 10.1186/s12888-026-08047-4 (PMC13188356; doi:10.1186/s12888-026-08047-4)
Supplement: Supplementary file 9 — Supplementary Material 9 [file 12888_2026_8047_MOESM9_ESM.docx]

Table S4. Quality Assessment Using the GRADE Framework of Each Pooled Analysis Assessing Associations Between PCOS Exposure and neuropsychiatric disorders outcomes in children.

| Outcomes | Studies, n | Study design | Risk of bias | Inconsistency | Indirectness | Imprecision | Publication bias | Plausible confounding | Magnitude of effect | Quality |
| --- | --- | --- | --- | --- | --- | --- | --- | --- | --- | --- |
| ASD | 10 | Observational | No serious limitations | No serious inconsistency | No serious indirectness | No serious imprecision | Undetected | No | No | Low |
| ADHD | 7 | Observational | No serious limitations | No serious inconsistency | No serious indirectness | No serious imprecision | Undetected | No | No | Low |
| CTD | 2 | Observational | No serious limitations | No serious inconsistency | No serious indirectness | Serious imprecision | Undetected | Yes | No | Very low |
| Anxiety | 3 | Observational | No serious limitations | No serious inconsistency | No serious indirectness | Serious imprecision | Undetected | Yes | No | Very low |
| ASQ domain (any fail) | 3 | Observational | No serious limitations | No serious inconsistency | Serious indirectness | Serious imprecision | Undetected | Yes | No | Very low |
| Other behavior/emotional problems | 1 | Observational | No serious limitations | NA | No serious indirectness | Serious imprecision | NA | Yes | No | Very low |
| Neurological malformations | 3 | Observational | No serious limitations | Serious inconsistency | No serious indirectness | Serious imprecision | Undetected | Yes | No | Very low |
